# Popeye sign of the semimembranosus

**DOI:** 10.1259/bjrcr.20170122

**Published:** 2018-04-30

**Authors:** Christopher Watura, Marcela De La Hoz Polo, Dimitri Amiras

**Affiliations:** 1 Department of Imaging, Mary’s Campus, Imperial College Healthcare NHS Trust, London, UK

## Abstract

A 23-year-old amateur football player presented 9 months after acute onset of severe pain and a lump in the posterior right knee whilst lifting a heavy box. He had been unable to return to playing football or climbing the stairs. Clinically, a Baker’s cyst was suspected. MRI scan, the imaging modality of choice, was essentially normal. A subsequent ultrasound (US) scan demonstrated abnormal dynamic bunching of the muscle fibres at the distal semimembranosus myotendinous junction on resisted isometric contraction, most likely due to a previous tear isolated to the distal myotendinous junction. The proximal biceps femoris tendon is the most commonly injured part of the hamstring. Distal semimembranosus tears are far less common. Semimembranosus tendinopathy is an uncommon cause of chronic knee pain that is probably underdiagnosed and inadequately treated. In this case, the distal semimembranosus injury was occult on MRI because the features were only apparent with dynamic imaging, something that is not routinely part of musculoskeletal MRI protocols, whereas real-time imaging is easily performed with US. MRI is thought to be more sensitive than US for follow-up imaging of healing hamstring injuries; however, this case highlights the usefulness of dynamic imaging of muscle injuries with US. We propose that the abnormal dynamic muscle bulge on the US image would be aptly described as a “Popeye sign,” which, to our knowledge, has not previously been reported in any other anatomical location than the long head of the biceps brachii in the published literature.

## INTRODUCTION

The semimembranosus, biceps femoris and semitendinosus muscles and their tendons make up the hamstring, which is located in the posterior compartment of the thigh. Among footballers, the long head of the biceps femoris and the proximal myotendinous junction (MTJ) of the semimembranosus are the more commonly injured hamstring components.^1^ In the general population, hamstring injury usually occurs at the proximal MTJ and most commonly affects the biceps femoris. Injury of the distal hamstring muscles is uncommon and usually affects the short head of the biceps femoris. Distal semimembranosus muscular tears are far less common.^2, 3^ MRI is the imaging modality of choice in detecting hamstring injury.^4^ The “Popeye sign” refers to an abnormal muscle bulge that enlarges on flexion, well known for obviating a tear of the long head of biceps brachii tendon.^5^

We present an unusual case of an amateur footballer who presented 9 months after his initial trauma. There was an isolated injury to the distal semimembranosus MTJ, which was occult on MRI but clearly demonstrated on a subsequent dynamic US scan, which showed an abnormal muscle bulge on flexion.

## CLINICAL PRESENTATION

**Figure 1.**
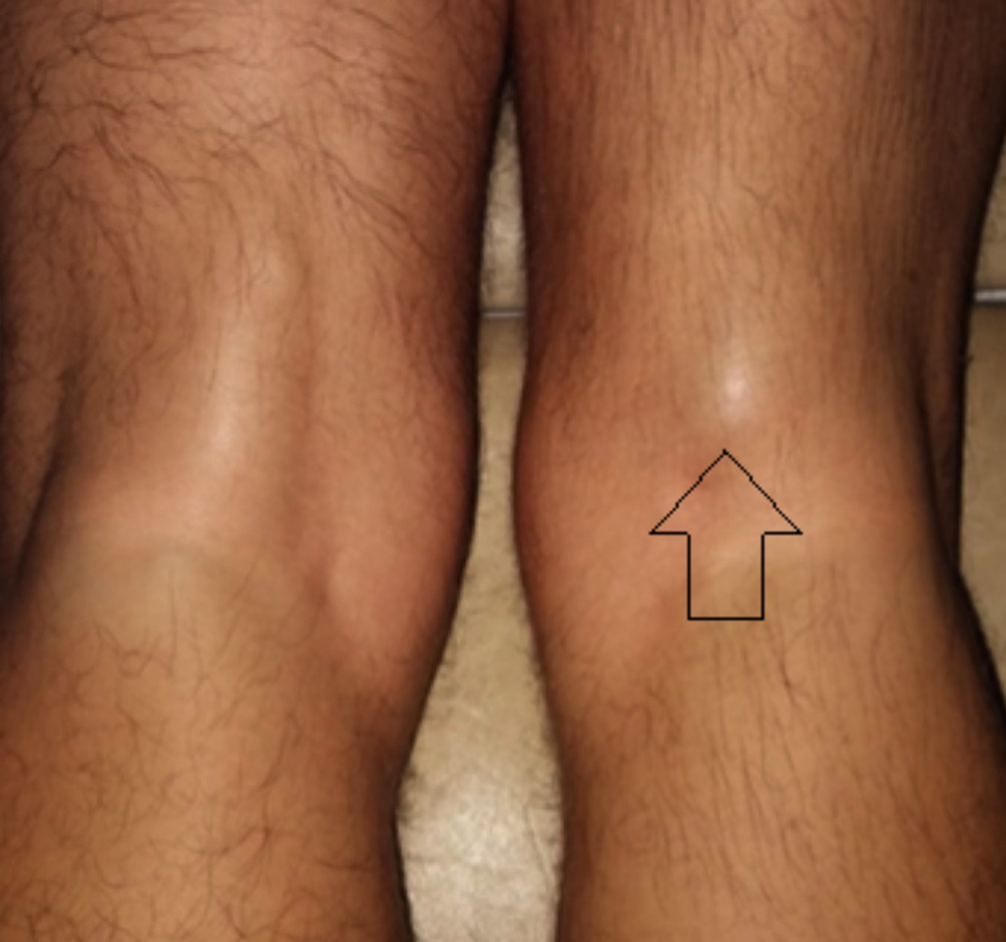
Photograph of the patient lying prone with isometric contraction of the affected limb (arrow). Note the normal appearance of the contralateral side on contraction.

A fit 23-year-old male, who played as a striker in amateur football, presented via his general practitioner with a lump in the posterior right knee. The patient described a 9-month history of right posterior knee pain associated with an intermittent lump that had come on suddenly whilst lifting a heavy box. This was the initial presentation, with no investigations prior. While the lump could be felt at rest, during resisted contraction it was much more easily palpable and became visible (Figure 1). Subjective pain severity was 6/10 and constant. He denied being able to play football, run or walk upstairs. Following clinical examination by his general practitioner, the main differential diagnosis was of a Baker’s cyst. The general practitioner referred the patient for MRI.

## DIFFERENTIAL DIAGNOSIS

- Baker’s cyst
- Muscle hernia
- Musculotendinous tear
- Soft tissue mass

## INVESTIGATIONS/IMAGING FINDINGS

**Figure 2.**
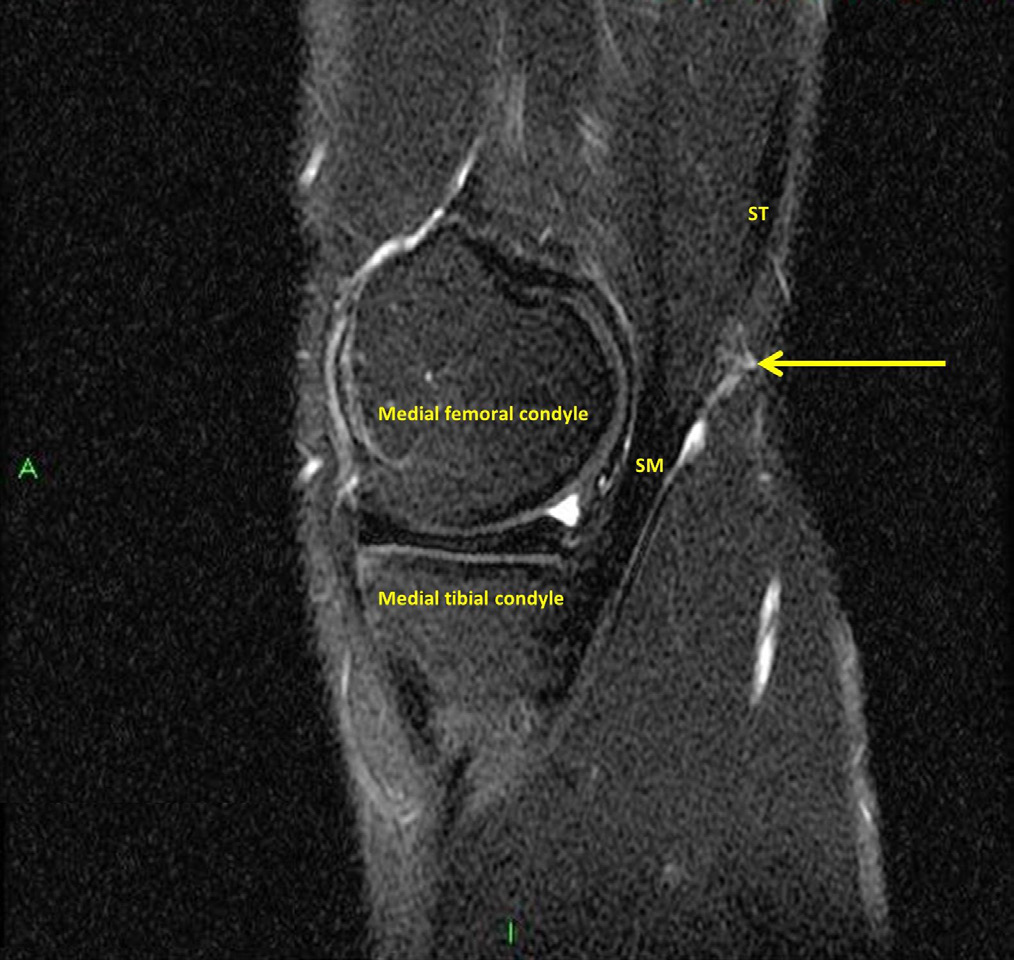
Sagittal proton density fat saturated MRI image demonstrating the intact semimembranosus muscle belly and distal tendon insertion. The arrow corresponds to the area of injury at the relatively long distal myotendinous junction adjacent to the bulky distal muscle belly.

**Figure 3.**
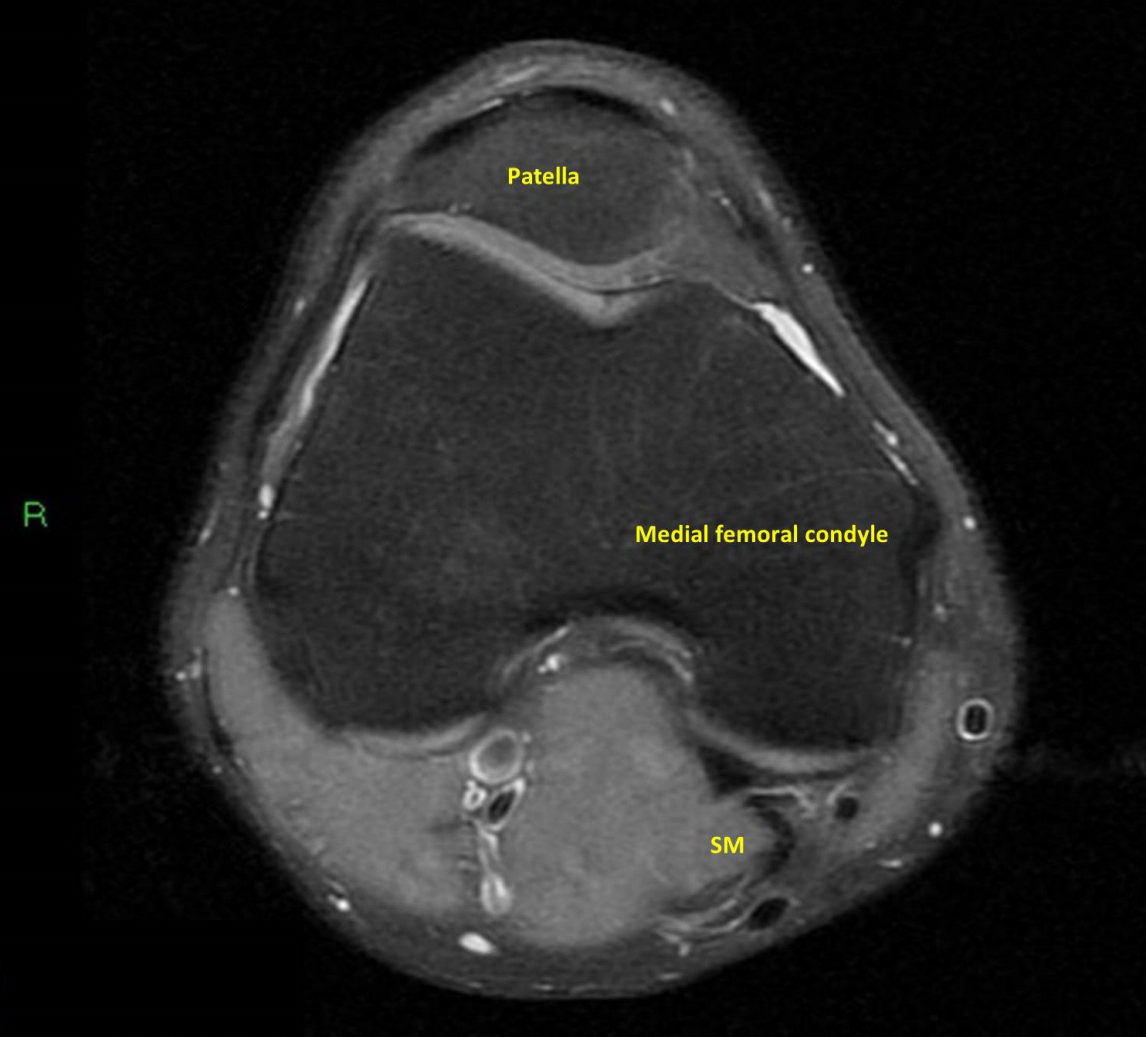
Axial proton density fat saturated MRI image corresponding to the area of semimembranosus musculotendinous injury. No abnormality is demonstrated.

MRI demonstrated no Baker’s cyst, no other posterior fossa mass and no definite cause for the pain or palpable lump (Figures 2 and 3). In particular, the semimembranosus and pes anserine tendons appeared normal, with no local sequelae of muscle injury such as oedema, contusion, haematoma or deformity. Following consultation with a sports medicine physician, a focused USund scan was performed by a consultant musculoskeletal radiologist.

**Figure 4.**
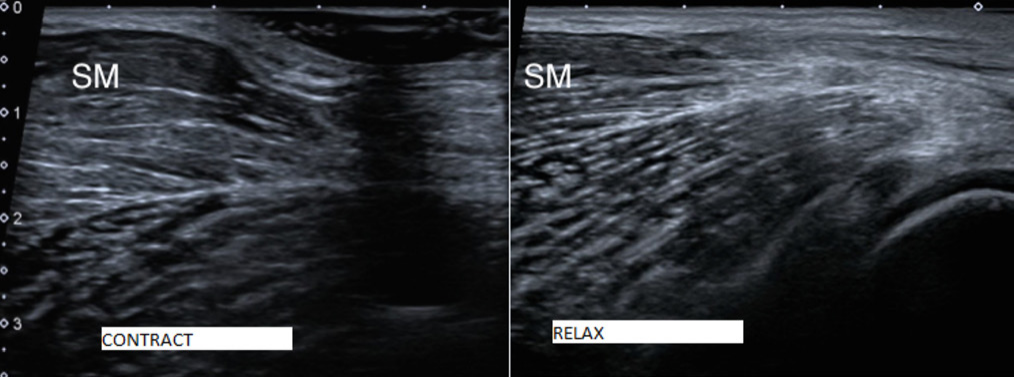
B-mode US image demonstrating bunching of muscle fibres at the distal MTJ of the semimembranosus on contraction (image on left). In each image, the distal portion of the MTJ is to the right. MTJ, myotendinous junction.

An Aplio 500 Toshiba US machine and linear 14 MHz transducer (Minato, Tokyo, Japan) were used for US scanning. When the transducer was placed longitudinally on the MTJ of the semimembranosus this was initially unremarkable. However, in real-time imaging there was abnormal dynamic bunching of muscle fibres at the distal semimembranosus MTJ during resisted isometric contraction. The appearances were most likely due to a previous tear isolated to the distal MTJ (Figure 4). While scanning, the observed muscle fibre bunching could be correlated with the appearance of the palpable lump felt by the patient.

## TREATMENT/OUTCOME

After 3 months of conservative treatment with physiotherapy, the patient returned to play without significant restriction. No follow-up imaging was considered necessary.

## DISCUSSION

**Figure 5.**
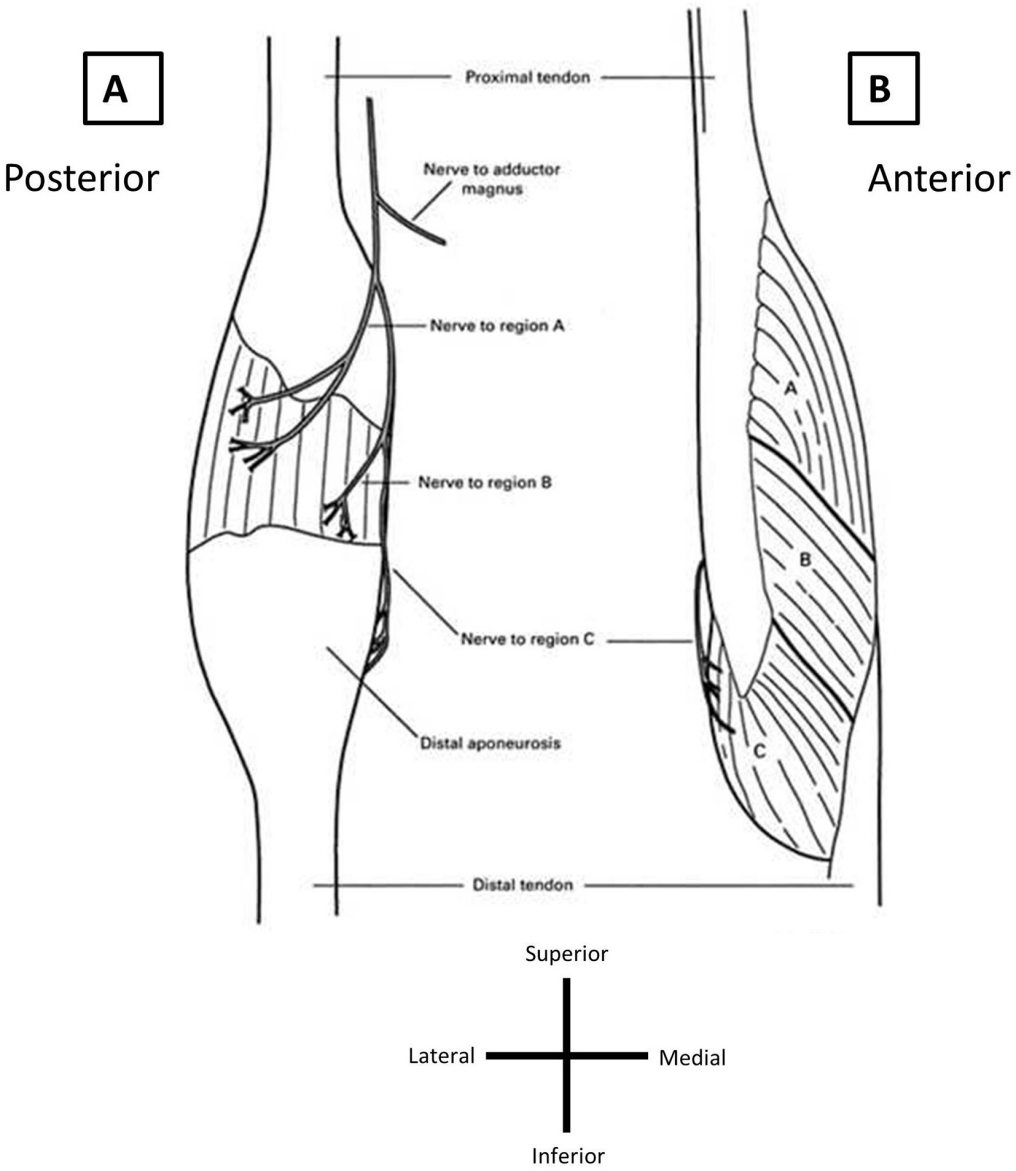
Adapted from Woodley et al. The semimembranosus has a bulky muscle belly, and three distinct regions are identified (regions a–c). The most distal fascicles arise from the thin medial surface of the proximal tendon (region c) and correspond with the injured muscle portion in this patient.

Semimembranosus is the largest of the hamstring muscles and originates from the lateral part of the ischial tuberosity, crossing deep to the semitendinosus and biceps femoris. It is a fusiform shaped muscle, the belly has a distinctive groove to accommodate the cord-like distal tendon of semitendinosus and is formed by three regions; the proximal two are unipennate and the distal one is thick and bipennate (Figure 5a–c). In comparison to the other hamstring muscles, its fascicles are the shortest (5 cm in length) and display the greatest pennation angle from the tendon, arranged as such for greater force production. Distally, the fascicles of semimembranosus insert into a large flat broad aponeurosis on the lateral side, which tapers to a short thick rounded tendon at its insertion into the posterior capsule of the knee and the posteromedial tibia approximately 1 cm distal to the knee joint line.^2^ The distal tendon (approximately 26 cm) is similar in length to that of semitendinosus and biceps femoris but the distal MTJ is the longest of all of the distal hamstring MTJs (approximately 19 cm). Semimembranosus tendinopathy usually affects the main head, reflected insertions or the distal tendon. During repetitive knee flexion, the distal semimembranosus tendon is subject to friction from the adjacent joint capsule, medial femoral condyle, medial tibial plateau and semitendinosus tendon. It is a combination of these anatomical features, friction forces and a propensity for eccentric contractions, which make the distal semimembranosus tendon relatively vulnerable to degenerative changes and strain injury.^6, 7^ Complete disruption of the MTJ usually results from sudden forceful contraction of a muscle against resistance. However, in many cases of distal semimembranosus tendon tear, there is a substantial degree of preceding degeneration.^2^ Semimembranosus tendinopathy is an uncommon cause of chronic knee pain that is probably underdiagnosed and inadequately treated owing to a lack of understanding of the condition.^7^

In a series of five patients with a distal semimembranosus MTJ tear, one complete and four partial tears, the patient with the complete tear was surgically treated 6 weeks after injury and was able to return to pre‐injury level of sports 6 months post-operatively. The patients with partial tears were operated on 6–72 months after injury and none of them were able to return to their pre‐injury level of sports. A muscle biopsy was performed in two of the four partial tear cases and this revealed signs of severe denervation of muscle fibres. This suggests good outcomes with early surgery for complete tears of the distal semimembranosus MTJ and that there is a risk of permanent muscle damage if a partial tear occurs. To our knowledge, there are no published results for conservative treatment.^8^

Our case describes an unusual presentation of a debilitating injury to the distal MTJ of the semimembranosus, most likely due to a previous partial tear. Unusually, the distal regions of the semimembranosus muscle were intact and the injury was isolated to the distal MTJ.^9^ MRI is the imaging modality of choice in detecting hamstring injury, although US is being increasingly used in elite sport and is readily accessible, cheap and dynamic.^4^ In the case we present, the injury features were occult on MRI because the abnormal bunching of muscle fibres at the distal semimembranosus MTJ was only apparent during resisted isometric hamstring contraction. There was no deformity at rest or local sequelae of muscle injury such as oedema, contusion or haematoma. Real-time imaging with US during isometric contraction of the hamstring was required to demonstrate the abnormal muscle bulge, thus allowing the correct diagnosis to be made. MRI is thought to be more sensitive than US for follow-up imaging of healing hamstring injuries.^10^ However, this case highlights the usefulness of real-time dynamic imaging with US in the follow-up of muscle injuries. In some scenarios, dynamic imaging with US may even be superior to MRI for differentiating musculotendinous pathologies, for example, muscle herniation versus tear. Making the correct diagnosis on US scanning was useful in directing physiotherapy and allowing the patient to understand the nature of the lump, which likely aided his return to playing football and daily activities such as climbing the stairs.

We suggest that the bunching of the semimembranosus muscle fibres on flexion creating a palpable bulge could be aptly described as a “Popeye sign” which, to our knowledge, has not been previously reported in any other anatomical location than the long head of the biceps brachii in the published literature.

## LEARNING POINTS

1. In patients presenting with a lump, the differential diagnosis of muscle fibre bunching following a tear should be considered, similar to a “Popeye sign” in biceps brachii tears.
2. Dynamic manoeuvres during real-time imaging with ultrasound can be used as a useful problem-solving adjunct to the gold standard of MRI in the diagnosis of musculotendinous injuries.
3. In some scenarios, dynamic imaging with US may even be superior to MRI for differentiating musculotendinous pathologies, for example, muscle herniation versus tear.
